# Effects of pravastatin, phytosterols, and combination therapy on lipid profile in HIV-infected patients: an open-labelled, randomized cross-over study

**DOI:** 10.1186/s13104-015-1225-6

**Published:** 2015-07-07

**Authors:** Noppadol Kietsiriroje, Rattana Leelawattana

**Affiliations:** Division of Endocrinology and Metabolism, Department of Internal Medicine, Faculty of Medicine, Prince of Songkla University, Songkla, 90110 Thailand

**Keywords:** Pravastatin, Phytosterols, Human immunodeficiency virus, LDL-c

## Abstract

**Background:**

To determine the effects of 40 mg of pravastatin, 2 g of phytosterols, and combination therapy on lipid profiles and to compare the reduction of LDL cholesterol between combination therapy and monotherapy.

**Methods:**

Thirty-six HIV-infected patients treated with ARVs who had high LDL cholesterol levels but no current usage of any lipid-lowering agents were enrolled into the open-labelled, randomized, cross-over study. All patients were assigned randomly into one of four intervention groups: (1) pravastatin 40 mg cross-over to the combination of pravastatin 40 mg and phytosterols 2 g (combination group), (2) the combination group cross-over to pravastatin 40 mg, (3) phytosterols 2 g cross-over to the combination group, and (4) the combination group cross-over to phytosterols 2 g. Each active treatment lasted 4 weeks with a wash-out period of 4 weeks.

**Results:**

The baseline mean TC, TG, HDL-c, and LDL-c levels in 36 HIV patients were 248.09 ± 34.73, 172.36 ± 125.44, 54.92 ± 16.67, and 175.13 ± 29.00 mg/dl, respectively. Pravastatin, phytosterols, and combination therapy reduced TC and LDL-c but TG and HDL-c were not significantly different from the baselines. The mean LDL-c reductions in the pravastatin, phytosterols, and the combination groups were 28.76 ± 9.32, 9.12 ± 7.84, and 27.08 ± 15.58%, respectively. The LDL-c levels in the pravastatin and combination groups were reduced more than in the phytosterols group (p < 0.01). There was no difference in the LDL-c reduction between the combination and pravastatin monotherapy groups (−25.61 ± 10.43 vs. −28.12 ± 14.07%, p = 0.555).

**Conclusion:**

Pravastatin had moderate potency on LDL-c lowering in HIV patients but could not bring LDL-c to goal. Adding phytosterols to pravastatin for a 4-week duration could not demonstrate any additional lipid-lowering effect

Trial registration: Thai Clinical Trial Registry: TCTR20150126002 date: January 23, 2015

**Electronic supplementary material:**

The online version of this article (doi:10.1186/s13104-015-1225-6) contains supplementary material, which is available to authorized users.

## Background

After the introduction of highly active antiretroviral therapy (HAART) together with a proper opportunistic infection prophylaxis regimen, HIV-infected patients (HIV patients) have a longer lifespan and better quality of life from a major reduction of AIDS-related mortality and morbidity. This improved longevity of HIV patients led to rising concerns about non-AIDS-related conditions [1], especially cardiovascular disease (CVD) [2]. One risk factor contributing to the increase in CVD among HIV patients using antiretroviral agents (ARVs) is an abnormal lipid profile. From the Data Collection on Adverse Events of Anti-HIV Drugs (DAD), the incidence of myocardial infarction increased with longer exposure to ARVs [3] and the prevalence of elevated total cholesterol (TC) (TC ≥240 mg/dL), low high-density lipoprotein cholesterol (HDL-c) (HDL-c ≤35 mg/dL) or elevated triglycerides (TG) (TG ≥204 mg/dL) also increased [4]. Mechanisms of abnormal lipid profile in HAART-treated HIV patients included increased de novo hepatic lipogenesis, increased secretion of very low-density lipoprotein (VLDL) and decreased clearance of VLDL, increased synthesis and decreased catabolism of apolipoprotein (Apo) B, and increased atherogenic low-density lipoprotein cholesterol (LDL-c) level including small dense LDL-c and oxidized LDL-c [5].

HMG-CoA reductase inhibitors or statins are the main agents to reduce the LDL-c level in most patients including HIV patients; however, more than 50% of HIV patients usually do not reach the LDL-c target defined by NCEP/ATP-III [6, 7]. The drug interaction via cytochrome P450 3A4 isozyme (CYP3A4) is the major concern in the use of HMG-CoA reductase inhibitors together with anti-retroviral agents (ARVs). The coadministration of simvastatin or lovastatin with protease inhibitors (PIs) is contraindicated [8]. Atorvastatin or rosuvastatin may be used in reduced dosages [8]. The most recent guideline suggests the use of pravastatin, atorvastatin, and fluvastatin to lower cholesterol in HIV patients but with the known pharmacokinetics of pravastatin was chosen mainly from its safety profile [8]. This unmet LDL-c target may be from the small dosage of potent statins used or the weak potency of an allowed statin to be used in patients taking HAART. Phytosterols, which are plant sterols/stanols and are classified as food, lower cholesterol by competitively binding with the Niemann-Pick C1-like 1 (NPC1L1) intestinal sterol transporter and lead to reduced gut cholesterol absorption. This mechanism of action is very similar to ezetimibe. In clinical experiments, when phytosterols are combined with statins there is an increase of LDL-c lowering efficacy of statins [9].Their effects on lipid metabolism have been studied and it was concluded that phytosterols are safe agents [10]. Thus, phytosterols are recommended as an LDL-c lowering agent in diet therapy in many guidelines [11–13]. However, there is no study report of their efficacy and safety in patients taking HAART.

This research aimed to study the efficacy of lipid-lowering treatment and short-term safety of a combination of phytosterols and pravastatin in HIV-infected patients with HAART.

## Methods

### Subjects

The subjects were HIV-infected patients recruited from the Infectious Clinic in the Outpatient Department of Songklanagarind Hospital from 22 November 2012 to 27 February 2014. All participants gave written informed consent. Inclusion criteria were HIV patients aged 20 years or older whose LDL-c was 130 mg/dL or higher at least 2 times in 3 months apart and the ARV regimen was unchanged within the previous 6 months. Exclusion criteria were active opportunistic infection, treatment with any lipid-lowering agents during the 2-month period before enrollment, hypersensitivity to any statins, and a transaminase enzyme level that was three times higher than the upper limit of normal.

### Study design

The medical ethics committee of Prince of Songkla University, Thailand approved the open-labelled, randomized, cross-over study. All participants were screened by coordinating nurses and all who met the criteria were randomized into one of four groups by a research coordinator using block randomization with a block size of four. Randomization was stratified by ages, sexes, and LDL-c levels. Only the investigators were blinded to the treatment allocation.

- Group 1. Pravastatin 40 mg cross-over to a combination group
- Group 2. Combination group cross-over to pravastatin 40 mg
- Group 3. Phytosterols 2 g cross-over to a combination group
- Group 4. Combination group cross-over to phytosterols 2 g

**Figure 1:**
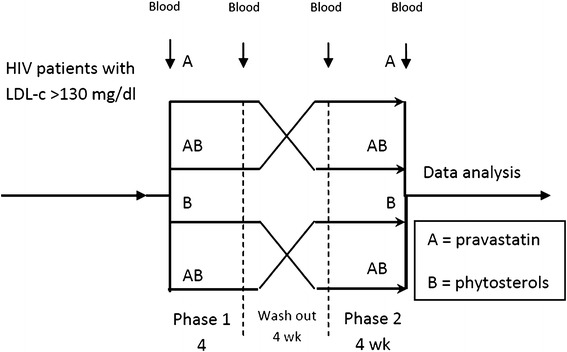
Diagram of cross-over study.

The flow of the trial is shown in Figure 1. In summary, the duration of the study was 12 weeks and was divided into 3 phases: 2 different active treatment phases separated by a wash-out phase. The medical history and physical examination of the patients taken at baseline included age, BMI, underlying diseases, duration of HIV infection, CD4 status and current ARVs. The biochemical tests at baseline and at the end of each intervention phase to determine the efficacy of each intervention included TC, TG, HDL-c, and LDL-c. Serum SGOT, SGPT, CPK, prothrombin time, and creatinine were collected at baseline and at the end of each intervention phase to determine the short-term safety of the interventions. Subject compliance was assessed by interviewing and counting residual pills and phytosterol products. Subjects who took pills or products less than 80% of the requirements were determined to be noncompliant. The subjects were asked to report any adverse effects at every follow-up time. All subjects were instructed to continue their usual lifestyle as well as dietary pattern.

### Pravastatin and phytosterol product

Pravastatin was taken after dinner once daily and phytosterols were taken in the form of a cereal beverage in the morning once daily. Tablets of 40 mg of pravastatin for the study were purchased from Daiichi Sankyo, EUROPE GmbH. The phytosterol product for the study was Hearti Benecol^®^ which contained 2 g of plant stanols per pack and was purchased directly from Sahapattanavibool Co., LTD., Thailand. A multivitamin preparation was the placebo for pravastatin with Nestvita^®^ (Nestle Thailand) which minimized an excess of calories from Hearti Benecol^®^ (Table 1). The nutritional facts of both products were provided by the manufacturers.

**Table 1:** Nutritional facts of phytosterol-enriched cereal beverage and standard cereal beverage

	Hearti Benecol^®^	Nestvita^®^
Total calories (kcal/1pack)	110	100
Total fat (g)	4	2
Total protein (g)	2	4
Total carbohydrate (g)	17	17
Fiber	1	4
Sugar	3	10
Total plant stanols	2.0	0
Plant stanol ester	1.15	

### Biochemical analysis

Venous blood samples were drawn between 8:00 a.m. and 10:00 a.m. after a 12-h fast. Serum aliquots were stored at room temperature and transported to the laboratory for testing on the same day. The fasting serum analyses of TC, TG, HDL-c, and LDL-c were measured by an enzymatic in vitro assay for direct quantification using a MODULAR P800 analyzer (Roche diagnostic, Switzerland). The intra- and inter-assay coefficient of variation to measure the parameters of total cholesterol, triglyceride, HDL-c, and LDL-c was less than 5%.

### Outcomes

The primary outcome was to compare the percent reduction of LDL-c of combination therapy to monotherapy. The secondary outcome was to determine changes of TC, TG, HDL-c, and LDL-c after the first phase of the pravastatin, phytosterols, and combination groups. The safety outcomes were any adverse events including nausea/vomiting, myalgia, increase in serum creatinine, myositis (defined as a CPK level more than five times the upper normal limit), and hepatitis (defined as a transaminase level more than three times the upper normal limit).

### Sample size calculation

The sample size was determined by simulation using a parallel group study design. LDL-c levels were assumed to have a normal distribution with a mean of 190 mg/dL and a standard deviation of 30. Three groups were created with these baseline parameters. For the after-treatment values, the data were duplicated to have mean LDL-c levels that were 10% lower for the group receiving phytosterols and 30% lower for the group receiving pravastatin.

For the group receiving the combination, the LDL-c levels were reduced by 50%, which was more than twice the additive effect of each drug alone. With these parameters, a dataset was created and a linear regression model was fit to the data and simulated 1,000 times. The number of simulations, which resulted in a significant p value for the interaction between the drugs and time, was recorded and the proportion determined as the power of the study. For a sample size of 20 persons per treatment arm, the power was calculated to be 88%. For a cross-over study, the authors assumed that half of the sample size would be adequate, that is, 10 patients per treatment arm. With an estimated drop-out rate of 20%, the total number of subjects required for a four-arm cross-over study was 48 patients. An interim analysis was done annually to determine the synergistic effect of the combination therapy on the lipid profile.

### Statistical analysis

The baseline characteristics were expressed as mean ± SD for parametric data, median and range for non-parametric data, and numbers and percent for categorical data. To determine the differences of baseline characteristics among the four study groups, one-way analysis of variance (ANOVA) was used for parametric data, the Kruskal–Wallis Test was used for non-parametric data, and the Likelihood Ratio test was used for categorical data.

To determine the primary outcome, paired Student’s t test was used to determine the mean difference of baseline LDL-c between the two intervention phases and the percent LDL-c reduction between monotherapy and combination therapy. To determine the secondary outcome, one-way ANOVA with least significant difference post hoc test correction was used. P values <0.05 were determined as statistically significant. R-3.1.2 for Windows was used for data analysis.

To determine the carryover effect for cross-over study design, mixed linear regression model with lm4 package in R program was used to test the effect.

## Results

**Figure 2:**
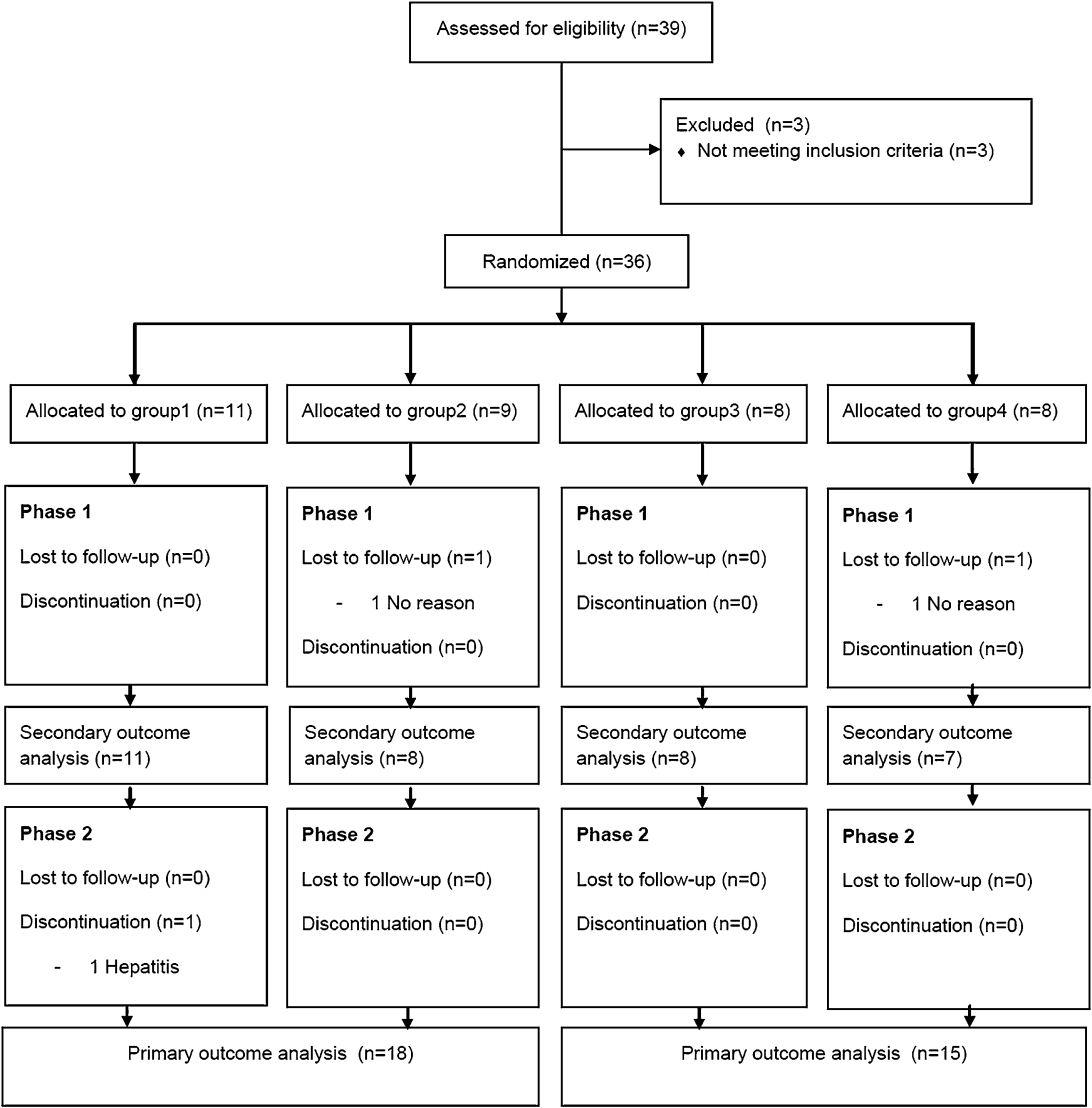
CONSORT flow diagram.

Thirty-nine HIV patients were screened and 36 HIV patients were enrolled into the study from 22 November 2012 to 27 February 2014. There were 34 subjects who completed the first phase and 33 patients who completed the second phase. Two subjects were lost to follow-up before the end of the first phase and one subject was excluded from the analysis due to adverse effects. Thirty-three subjects who completed the second phase were included into the primary outcome analysis and 34 subjects who completed the first phase were included into secondary outcome analysis (Figure 2). The baseline characteristics of each treatment group are summarized in Table 2. There were no differences in the baseline characteristics among the 4 treatment groups. The mean age of all patients was 44.68 ± 7.4 years old, mean BMI was 22.60 ± 2.83 kg/m^2^, mean HIV duration was 7.91 ± 5.4 years, and the mean CD4 status was 561.47 ± 316.10 cells/cmm. Of the 36 patients, 63.9% were male. One subject had diabetes, one subject had hypertension, four subjects were using PIs and three subjected had unsuppressed HIV viral load. The baseline means of the TC, TG, HDL-c, and LDL-c levels in the 36 HIV patients were 248.09 ± 34.73, 172.36 ± 125.44, 54.92 ± 16.67, and 175.13 ± 29.00 mg/dl, respectively. The baseline LDL-c levels in Groups 1–4 were 172.76 ± 43.23, 187.78 ± 26.46, 162.80 ± 11.29, and 180.68 ± 41.86 mg/dl, respectively. One subject was determined as noncompliant.

**Table 2:** Baseline characteristics of subjects

	Mean ± SD or median (min,max) or number (%)				p value
	Group 1 (n = 11)	Group 2 (n = 9)	Group 3 (n = 8)	Group 4 (n = 8)	
Age, year	47.94 ± 7.73	45.33 ± 8.66	41.12 ± 7.72	43.00 ± 7.41	0.23^b^
BMI, kg/m^2^	22.48 ± 2.40	22.68 ± 3.64	23.63 ± 3.07	21.61 ± 2.45	0.59^b^
Male, no.	8 (72.7)	7 (77.8)	4 (50)	4 (50)	0.48^c^
Diabetes, no.	0	1 (11.1)	0	0	0.41^c^
Hypertension, no	1 (9.1)	1 (11.1)	0	0	0.48^c^
PIs, no.	2 (18.2)	1 (11.1)	0	1 (12.5)	0.50^c^
NNRTI, no.	9 (81.8)	8 (88.9)	8 (100)	7 (87.5)	
Suppressed HIV viral load, no^d^	9 (81.8)	8 (88.9)	8 (100)	8 (100)	0.27^c^
HIV duration, years	5.61 ± 5.21	11.11 ± 3.79	6.61 ± 4.51	8.78 ± 7.11	0.13^b^
CD4, cell/cmm	514.27 ± 331.71	678.22 ± 438.85	612.62 ± 270.87	443.88 ± 152.96	0.45^b^
Creatinine, mg/dL	0.90 ± 0.19	0.95 ± 0.28	0.94 ± 0.35	0.73 ± 0.13	0.29^b^
SGPT, mg/dL	35.81 ± 24.46	35.66 ± 15.02	30.13 ± 12.77	27.50 ± 8.18	0.67^b^
HDL-c, mg/dL	52.07 ± 13.23	53.04 ± 12.08	57.73 ± 20.93	58.13 ± 23.15	0.83^b^
LDL-c, mg/dL	175.75 ± 36.90	186.99 ± 24.60	163.01 ± 9.65	173.04 ± 35.24	0.43^b^
TC, mg/dL	222.0 (197,301)	245.0 (227,353)	242.5 (191,285)	242.0 (202,297)	0.48^a^
TG, mg/dL	134.0 (64,390)	176.00 (53,620)	106.50 (58,499)	109.0 (52,367)	0.54^a^
CPK, mg/dL	131.0 (46,876)	150.0 (87,234)	133.5 (60,289)	123.0 (79,532)	0.96^a^

Group 1, pravastatin then combination; Group 2, combination then pravastatin; Group 3, phytosterols then combination; Group 4, combination then phytosterols.

^a^Kruskal–Wallis Test.

^b^One-way ANOVA.

^c^Likelihood Ratio test.

^d^Suppressed HIV viral load defined by HIV viral load <50 copies/ml.

**Figure 3:**
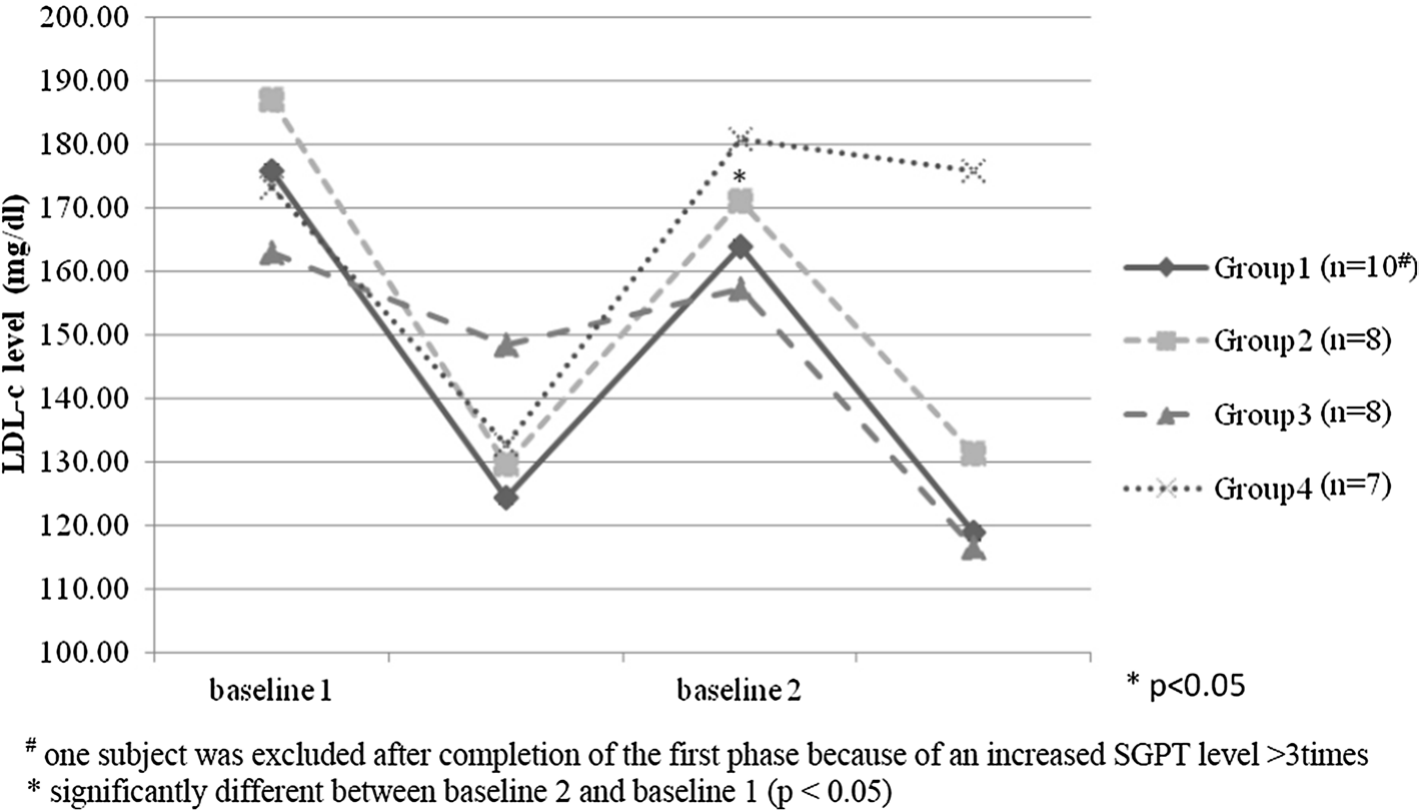
Baseline of serum LDL-c of phase 1 and phase 2 of each group. Baseline 1, baseline LDL-c level before the start of active treatments in phase 1; baseline 2 LDL-c level after the washout period; Group 1, pravastatin then combination; Group 2, combination then pravastatin; Group 3, phytosterols then combination; Group 4, combination then phytosterols.

The changes in the LDL-c levels of the 4 groups are shown in Figure 3. After the wash-out period, the LDL-c levels returned to similar baseline levels of the first phase (data not shown) except in Group 2 in which the second baseline was slightly but significantly lower than the first baseline. (186.19 ± 26.18 vs. 170.95 ± 32.31 mg/dL, p = 0.042). The statistical difference of carryover effect was not observed in this study (Additional file 1).

**Figure 4:**
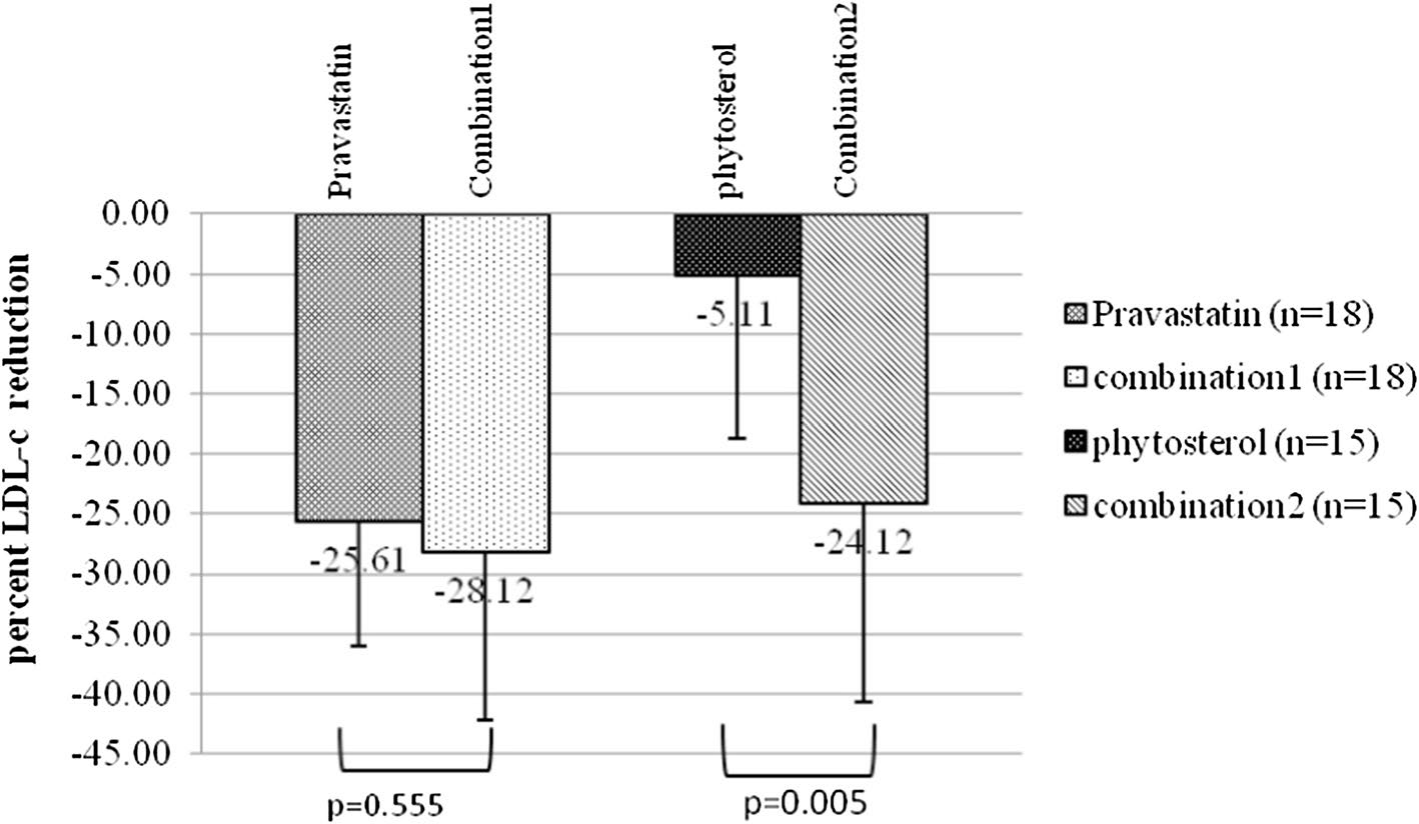
Comparison of LDL-c reduction between combination therapy and monotherapy. Pravastatin pravastatin, monotherapy in Groups 1 and 2; combination 1, combination therapy in Groups 1 and 2; phytosterols, phytosterols monotherapy in Groups 3 and 4; combination 2 combination therapy in Groups 3 and 4.

The data from Groups 1 and 2 were used to determine the difference of LDL-c reduction between the pravastatin monotherapy and the combination therapy while the data from Groups 3 and 4 were used for phytosterols monotherapy and the combination therapy. A comparison of the LDL-c reduction efficacies between each monotherapy and combination therapy are shown in Figure 4. There were no differences of LDL-c reduction between the combination therapy and pravastatin monotherapy (25.61 ± 10.43 vs. 28.12 ± 14.07%, p = 0.555). The LDL-c reduction of combination therapy was better than phytosterol monotherapy (24.12 ± 16.56 vs. 5.11 ± 13.66%, p = 0.005).

The lipid reducing efficacies of pravastatin, phytosterols, and combination therapy were determined after 4 weeks of the first phase intervention and the results are shown in Table 3. The decreased mean percentages of TC in the pravastatin, phytosterols, and combination therapy groups were 17.79 ± 5.44, 4.16 ± 7.60, and 17.70 ± 12.64, respectively, and the decreased mean percentages of LDL-c were 28.76 ± 9.32, 9.12 ± 7.84, and 27.08 ± 15.58, respectively, (pravastatin or the combination therapy compared to phytosterols, p < 0.01). The TG levels remained unchanged in all treatment groups although there was a small decrease in the pravastatin treatment group. However, the magnitude of change was not statistically significant. The changes in the HDL-c showed some similarities to that of TG; there was some nonsignificant rising of HDL-c in the pravastatin group, whereas in the phytosterols or combination group there were no substantial changes in the HDL-c levels.

**Table 3:** Percent change of lipid parameters in each treatment group at the end of the first phase

Lipid parameters	Pravastatin (n = 11)		Phytosterols (n = 8)		Combination (n = 15)		p value
	Mean	SD	Mean	SD	Mean	SD	
TC	−17.79	5.44	−4.16	7.60	−17.70	12.64	0.006*
TG	−16.33	29.82	−4.08	23.98	−0.52	42.09	0.516
HDL-c	5.27	8.47	−0.07	14.07	−2.31	12.97	0.290
LDL-c	−28.76	9.32	−9.12	7.84	−27.08	15.68	0.003*

* Significantly different between either pravastatin or combination compared to phytosterols group (p = 0.002).

**Figure 5:**
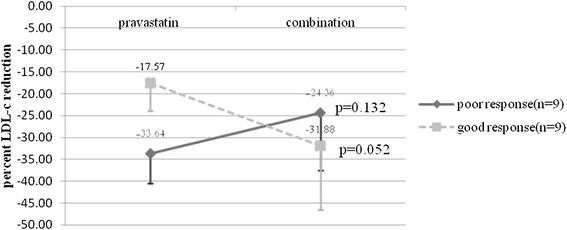
Effects of phytosterols on patients who had good response and poor response to pravastatin. Pravastatin, pravastatin monotherapy in Groups 1 and 2; combination combination therapy in Groups 1 and 2; poor response, subgroup pravastatin with an LDL-c reduction less than 25.6%; good response, subgroup pravastatin with an LDL-c reduction more than 25.6%.

When the HIV patients were separated according to the reduction of LDL-c by pravastatin into good and poor response using the mean value of 25.6%, a trend of interaction was demonstrated between the addition of phytosterols and the responsiveness to pravastatin. Phytosterols reduced LDL-c further from a mean of 17.54 ± 6.30 to 31.88 ± 14.68% (p = 0.052) in the poor response group while adding phytosterols to the good response patients possibly interfered with the LDL-c reducing efficacy of pravastatin (33.64 ± 6.81 to 24.36 ± 13.16% (p = 0.132) (Figure 5).

### Safety

One subject had an elevated serum transaminase enzyme just above 3 times of the upper normal limit after receiving pravastatin monotherapy. However, this subject had active hepatitis B with a twofold elevated transaminase enzyme at baseline. His transaminase enzyme level declined to his baseline after withdrawal of pravastatin.

## Discussion

To our knowledge, this is the first study to determine the effects of a combination of pravastatin and phytosterols to lower LDL-c in HIV-infected patients taking HAART. Many guidelines recommend rosuvastatin, fluvastatin, and pravastatin as the statins of choice in HIV-patients. These statins do not interfere with ARVs and the recommended initial dosage of pravastatin is 40 mg/day. In the general population, 40 mg of pravastatin showed an efficacy in LDL reduction of 29.7% [14]. The efficacy of 40 mg of pravastatin on LDL reduction in HIV-infected patients in placebo-controlled trials was approximately 20% [15, 16]. Head-to-head studies compared the efficacy of different statins, including 10 mg of rosuvastatin and 10 mg of atorvastatin among HIV-infected patients who received protease inhibitors. These studies also confirmed that the LDL-reduction efficacies of 20 mg and 40 mg of pravastatin were 17.6 and 19%, respectively [17, 18]. During the first phase, the data confirmed the efficacy of 40 mg of pravastatin in those patients taking HAART. The LDL-c reduction efficacy of pravastatin was approximately 29%, which was similar to the non-HIV infected patients from a previous study [14] and seemed to be better when compared to studies among HIV-infected patients. This may be because the proportion of PI used in this study was very low compared to previous studies [15, 16, 18].

The LDL-c reduction efficacy of phytosterols in this study was 9.12% which was similar to a previous study in the Thai population which showed a 8.9% LDL-c reduction [19]. The effect of phytosterols on LDL-c reduction was modest (8–14%) in accordance with previous studies [20–22]. One meta-analysis reported that the efficacy of 2 g/day of plant stanols or sterols was a reduction in the LDL-c levels by 10% [23]. The effect of phytosterols on triglyceride levels and HDL-c was very little, which was similar to one meta-analysis that reported no effect of phytosterols on triglyceride and HDL-c levels [9]. The ESC/EAS guideline also reported that 2 g daily of phytosterols reduced total cholesterol and LDL-c by 7–10% with little or no effect on TG and HDL-c when it was consumed with the main meal [24]. Thus, the effectiveness of a phytosterol product on the lipid profiles in this study was similar to previous reports.

The LDL-c reduction efficacy of the combination therapy was 28% which was similar to pravastatin monotherapy. With this cross-over design, there was no difference in percent changes of LDL-c levels between the combination therapy and pravastatin alone. It was surprising that the effect of phytosterols in lowering LDL-c was not observed when the phytosterol product was combined with pravastatin. The possibilities could be from the short duration of statin treatment, low rates of cholesterol absorption in the patients, or compliance issues of these vulnerable subjects.

The effect of duration of statin treatment might play a major role on the effect of phytosterols. The mechanism of action of HMG-coA reductase inhibitor includes the inhibition of hepatic LDL-c synthesis, which leads to upregulation of LDL-R expression and leads to a compensatory increase of cholesterol absorption from the intestine [25, 26]. The action of phytosterols should be seen clearly only if the intestinal cholesterol absorption is substantial. A meta-analysis on the effect of adding plant sterols or plant stanols to statins on LDL-c showed that add-on phytosterols to statin therapy was better than statin alone [9]. However, most of the included trials studied patients who took a steady dosage of statin for at least 8 weeks before adding phytosterols. However, a study by Gylling et al. [22] also enrolled naïve patients and showed a benefit of combining phytosterols with statin therapy. The difference between this study and the Gylling study was the duration of the active phase. The patients in this study were on active treatment for only 4 weeks each. In the Gylling study, the duration of each treatment phase was 7 weeks. However, Simons and colleagues demonstrated the additive effect of approximately 6% of add-on phytosterols to cerivastatin sodium when the subjects started simultaneously for a duration of 4 weeks [20]. The difference in this study from the Simons study was the statin used. Cerivastatin is a potent statin and the effect of NPCL1 inhibition on LDL-c reduction is more pronounced [25]. An additional LDL-c lowering effect of phytosterols on statin therapy was not demonstrated in this study possibly because the dietary patterns and other changes during the study were not monitored.

From the subgroup analysis of this study, the poorer effect of phytosterols on good responders indicated another hypothesis concerning the differentiation of synthesis and absorption of cholesterol. Phytosterols would have an additive effect on LDL reduction only in patients with low rates of cholesterol synthesis and high rates of cholesterol absorption. The poor responders of the pravastatin subgroup would be the ones with low rates of cholesterol synthesis and high rates of cholesterol absorption and in this group of patients, the phytosterols should exhibit their maximal effect.

This study reported good compliance of most subjects and apparent compliance in counting the residual pills and products. However, there was no objective protocol to assure real compliance as these patients also had to take a large number of drugs. However, from the accepted LDL-c reduction between the pre- and post-interventions of each phase, compliance of the subjects was not a major problem.

The limitations of this study were the small sample size and the unblinded fashion of the study design of the patients. The small sample size was not unexpected among this kind of vulnerable patients. They used multiple medications and were not willing to participate in a prevention trial which could not demonstrate any beneficial effect. To correct this limitation, a multicenter study could be the solution. Another limitation was the open-labelled study design. The patients knew the products and medicines, which possibly affected their behavior and diet. For example, in the group that had the non-phytosterol-enriched product possibly tried to control their behavior while the other group that had the phytosterol-enriched product possibly had poorer food choices. Since this present study did not design for dietary control and monitoring, this confounder could not be demonstrated.

One of the strengths of this study is that it is the first study that used phytosterols to reduce LDL-c in HIV patients (Additional file 2). The other strength was the emphasis on the recommended diet by standard guidelines in which 2 g of phytosterols could reduce LDL-c level in HIV patients.

In this study, pravastatin was safe for patients taking HAART. Only one patient had asymptomatic elevated transaminase enzyme but that patient also had chronic active hepatitis virus B.

## Conclusion

Pravastatin had moderate potency on lowering the LDL-c levels in HIV patients but could not bring LDL-c to goal. Adding phytosterols to pravastatin for the 4-week duration could not demonstrate any additive lipid-lowering effect.

## Additional files

**Additional file 1.** Testing carryover effect on LDL-c outcome.
**Additional file 2.** CONSORT 2010 checklist of information to include when reporting a randomised trial.

## Abbreviations

AIDS: acquired immune deficiency syndrome
Apo: apolipoprotein
ARVs: antiretrovival agents
CPK: creatinine phosphokinase
CVD: cardiovascular disease
CYP: cytochrome P450
HAART: highly active antiretroviral therapy
HDL-c: high-density lipoprotein cholesterol
HIV: human immunodeficiency virus
LDL-c: low-density lipoprotein cholesterol
NPC1L1: Niemann-Pick C1-like 1
PIs: protease inhibitors
SGPT: serum glutamic-pyruvic transaminase)
SGOT: serum glutamic oxaloacetic transaminase
TC: total cholesterol
TG: triglyceride
