# Supplementary material for: Effects of pravastatin, phytosterols, and combination therapy on lipid profile in HIV-infected patients: an open-labelled, randomized cross-over study
Source: BMC Res Notes. 2015 Jul 7;8:294. doi: 10.1186/s13104-015-1225-6 (PMC4492082; doi:10.1186/s13104-015-1225-6)
Supplement: Supplementary file 1 — Additional file 1. Testing carryover effect on LDL-c outcome. [file 13104_2015_1225_MOESM1_ESM.docx]

**Testing carryover effect on LDL-c outcome**

Design: Crossover design

4 sequence (AR; RA; BR; RB)

A= Pravastatin 40 mg; B= Phytosterols 2 g; R=Reference (combination group)

Period: 2; Drug: A, B, R

Response to reviewer (carryover effect on LDL-c)

We tested the carryover effect between Drug A and Combination and Drug B and Combination. Mixed linear regression model with lm4 package in r program was used to test the effect. We found that there was no statistical difference of carryover effect between sequence AR and RA (P value: 0.977). The statistical difference of carryover effect between sequence BR and RB was not observed as well (P value: 0.659). So in this study we could conclud that no carryover effect was observed.

Statistical modeling for testing carryover effect as follow:

$y_{ijk}=\mu+b_{ij}+\pi_{k}+\phi_{m}+\lambda_{m}+\varepsilon_{ijk}$	Equation (1)

Where i = sequence, j = patient, k = period and m = treatment

$\mu$ : Overall mean

$b_{ij}$: Effect of j^th^ patient with i^th^ sequence

$\pi_{k}$: Effect of k^th^ period

$\phi_{m}$: Effect of m^th^ drug treatment

$\lambda_{m}$: Residual effect of m^th^ drug treatment

$\varepsilon_{ijk}$: Random error

We tested two hypothesis of carryover effect that is AR|RA and BR | RB.

Hypothesis I, we tested for sequence AR|RA (group 1 vs 2)

Ho: there was no difference average of ldl between sequence AR and RA ($b_{ij}$ = 0)

Ha: there was difference average of ldl between sequence AR and RA ($b_{ij}$ ≠ 0)

Hypothesis II, we tested for sequence BR|RB (group 3 vs 4)

Ho: there was no difference average of ldl between sequence BR and RB ($b_{ij}$ = 0)

Ha: there was difference average of ldl between sequence BR and RB ($b_{ij}$ ≠ 0)

If P value of sequence from modelling was no statistical significant (P value >0.05), we could concluded that no carryover effect.

Table 1 show the effect of carryover among sequence (AR | RA, BR | RB)

|  | Coefficient | SE | t value | p-value |
| --- | --- | --- | --- | --- |
| LDL (outcome) |  |  |  |  |
| $b_{ij}$ : Sequence (AR vs RA) | 0.25 | 8.53 | 0.029 | **0.977** |
| $\pi_{k}$ : Period (1 vs 2) | 11.73 | 8.53 | 1.375 | 0.169 |
| $\phi_{m}$: Drug (A vs R) | -5.18 | 8.53 | -0.607 | 0.544 |
|  |  |  |  |  |
| LDL (outcome) |  |  |  |  |
| $b_{ij}$ : Sequence (BR vs RB) | 4.28 | 9.69 | 0.442 | **0.659** |
| $\pi_{k}$ : Period (1 vs 2) | 5.41 | 9.46 | 0.572 | 0.567 |
| $\phi_{m}$: Drug (B vs R) | 31.59 | 9.46 | 3.339 | 0.001 |

From table 1, the sequence effect was not statistical difference both sequence AR|AR (P value = 0.977) and BR | RB (P value=0.659).

From this graph, we seen that, at the period II sequence 4 (baseline 2, group 4), the value of LDL-c level was higher than baseline 1. It might be that this results occurred by participant status or a tendency of participants to have a high LDL-c level after period 1.

Reference

Dieter H, Volker S, Iris P. 2007. Bioequivalence Studies in Drug Development: Methods and Applications. 1^st^. New York: John Wiley & Sons Inc
